# Delays in diagnosis and treatment of depressive disorder among young adults: A national online survey-based cross-sectional study

**DOI:** 10.1371/journal.pone.0351402

**Published:** 2026-06-12

**Authors:** Rena Xu, David Pletta, Caitlynn Feng, Cassandra Morrow, Maya C. Clark, Badar Omar, Alex S. Keuroghlian, Sari L. Reisner

**Affiliations:** 1 Department of Urology, Boston Children’s Hospital, Boston, Massachusetts‌‌, United States of America; 2 Department of Surgery, Harvard Medical School, Boston, Massachusetts, United States of America; 3 Department of Epidemiology, University of Michigan School of Public Health, Ann Arbor‌‌, Michigan, United States of America; 4 Department of Psychiatry, Massachusetts General Hospital, Boston, Massachusetts, United States of America; 5 Department of Psychiatry, MaineHealth, Portland, Maine, United States of America; University of Malaga: Universidad de Malaga, SPAIN

## Abstract

**Background:**

Depression is highly prevalent among U.S. young adults and associated with long-term functional impairment and increased suicide risk. While delays in diagnosis and treatment of depression are well documented among older adults, the magnitude and predictors of such delays in the younger population are poorly understood.

**Objective:**

To characterize the time to diagnosis and treatment of depressive disorder and predictors of diagnostic and treatment delay among young adults.

**Methods:**

This cross-sectional study used a self-reported survey conducted via the online research platform Prolific in May 2025. Eligible participants were U.S. adults aged 18–35 years with a history of at least one depressive episode. Sociodemographic, clinical, and psychosocial characteristics, including age of depressive symptom onset, degree of social support, and frequency of social group engagement, were assessed. Primary outcomes were probability of not receiving a depressive disorder diagnosis despite symptoms, time from symptom onset to diagnosis, probability of not seeking treatment, and time from symptom onset to treatment. Secondary outcomes were perceived treatment effectiveness and current symptom control.

**Results:**

In total, 871 respondents met inclusion criteria. Of those with one or more lifetime depressive episodes, 46.2% reported never receiving a depressive disorder diagnosis. Median time from symptom onset to diagnosis was 3 years (IQR: 0–7). Over a quarter (27.4%) never sought treatment; among those who did, 93.5% received care, but 31.4% experienced a delay of 1–4 years, and 28.8% experienced a delay of 5 + years. Symptom onset in childhood (ages 0–12) or adolescence (ages 13–17) was associated with longer time to diagnosis and treatment and lower perceived treatment effectiveness. Greater social support was associated with shorter time to diagnosis; lower probability of never receiving a diagnosis, never seeking treatment, or experiencing prolonged treatment delay; and higher perceived treatment effectiveness and current symptom control. Frequent engagement in social groups was also associated with greater perceived treatment effectiveness.

**Conclusions:**

Among U.S. young adults, prolonged delays in depression diagnosis and treatment are common. Early symptom onset is associated with longer delays and worse outcomes, whereas greater social support is associated with shorter delays and more favorable outcomes. These findings highlight the need for further research to clarify causal mechanisms and for interventions to promote timely diagnosis and treatment among young adults at risk for depression.

## Introduction

Depression is a growing problem among young adults in the United States. Each year, more than 15% of U.S. adolescents aged 12–17 years experience a major depressive episode [1], and emerging adults aged 18–25 years have the highest incidence of depression of any age group [2]. As rates of depression rise among young people [3], the gap between prevalence and treatment is widening [4]. Delays in diagnosis and treatment may contribute substantially to this gap. Among adults, the average delay from symptom onset to treatment of mood disorders has been estimated at six to eight years [5], and longer delays are strongly associated with worse outcomes [6]. For younger populations, the magnitude and predictors of diagnostic and treatment delay remain poorly understood. Early symptom onset has been linked to longer illness duration and greater severity in bipolar disorder, but its impact on timeliness of care for other depressive disorders has not been well characterized [7].

Compounding these mental health challenges is an epidemic of loneliness, with rates of loneliness rising steadily since the 1970s [8]. Social isolation is a risk factor for suicidality, whereas social support buffers against depression onset and improves outcomes among individuals with known depressive disorders [9–12]. Despite these well-established associations, the impact of social support on diagnostic and treatment trajectories is not well defined.

This study aimed to characterize the prevalence and duration of diagnostic and treatment delays for depressive disorders among U.S. young adults and identify demographic, clinical, and psychosocial factors associated with these delays. The study was conducted as part of a National Institutes of Health (NIH)-funded project (U01MH136558) to develop and implement innovative interventions to improve mental health outcomes for young adults at high risk for depression and suicidality. The purpose of this research inquiry was to inform intervention development and deployment by elucidating the challenges that young adults face in receiving appropriate diagnoses and treatment for depressive disorder, delineating risk factors, and identifying modifiable factors that may be associated with such delays. We hypothesized that earlier symptom onset would be associated with greater delays and lower treatment effectiveness, whereas greater social support would be associated with shorter delays and more favorable outcomes.

## Materials and methods

### Study design

We collected data using a national cross-sectional, self-reported electronic survey conducted via the online research platform Prolific between May 9, 2025, and May 30, 2025.

### Participants

Eligible participants were U.S. adults aged 18–35 years who could provide informed consent and complete the survey in English and who reported at least one lifetime depressive episode or period of depression symptoms lasting 2 weeks or longer. Participants also had to provide the age at which they first experienced depressive symptoms. We selected the age range of 18–35 years to encompass both emerging adulthood (ages 18–29) and early adulthood (ages 30–35). A random sample of prospective participants meeting eligibility criteria based on Prolific profile data were invited by the platform to participate. All eligibility criteria were confirmed within the survey.

### Ethical considerations

Study procedures were approved by the Institutional Review Board at Boston Children’s Hospital (IRB-P00049962). All participants provided electronic written informed consent before participation and were compensated $3 for survey completion. All data were collected anonymously. No minors participated in the study. This study followed the Strengthening the Reporting of Observational Studies in Epidemiology (STROBE) guidelines. A completed STROBE checklist is provided as Supporting Information (S1 Appendix) [13].

### Measures

#### Outcomes‌‌.

Primary outcomes of interest were: 1) probability of not receiving a depressive disorder diagnosis despite symptoms, 2) time from symptom onset to diagnosis, 3) probability of never seeking treatment, and 4) probability of treatment delay (defined as ≥1 year from symptom onset to first treatment). Secondary outcomes were perceived effectiveness of lifetime treatment and control of current symptoms among participants receiving active treatment. Survey measures were anchored in Diagnostic and Statistical Manual for Mental Disorders, Fifth Edition, Text Revision (DSM-5-TR) criteria for major depressive disorder and major depressive episode or adapted from prior validated research [5,14–18]. Current depressive symptoms were assessed using the Patient Health Questionnaire-4 (PHQ-4), a validated ultra-brief screening instrument [19]. Lifetime history of depressive episodes was assessed via an item that asked, “Did you ever in your life have a depressive episode or depression symptoms (e.g., a period of low or depressed mood such as feeling sadness, despair, irritable, empty, or loss of interest or pleasure in activities that lasted most of the day nearly every day for at least 2 weeks)?” History of diagnosed depressive disorders was assessed using a series of questions about diagnoses received from medical or mental health professionals.

#### Demographic factors.

Age was recorded as a continuous variable and categorically coded (18–22, 23–25, 26–29, and 30–35 years) to reflect stages of emerging and early adulthood. Other demographic variables included race, gender identity, sexual orientation, education level, employment status, and community type (urban, suburban, rural, or other). Participants self-reported their state of residence, which was coded by U.S. census region (Northeast, Midwest, South, or West).

#### Clinical and psychosocial factors.

General health was assessed using an item from the 12-Item Short Form Health Survey (SF-12) and dichotomized as “poor/fair” vs. “good/very good/excellent” [20]. Psychiatric comorbidities were assessed using a checklist of 10 common non-depressive disorders (e.g., anxiety) with a write-in option for other diagnoses. Responses were summed and categorized as 0, 1, or 2 + comorbidities. Childhood adversity was assessed using four items from the household dysfunction domain of the Adverse Childhood Experiences (ACEs) questionnaire: parental separation or divorce, household substance use, household mental illness, and household member incarceration [21]. These were summed and similarly categorized as 0, 1, or 2 + . Items were selected to capture household-level adversity that may shape pathways to diagnosis and treatment. Items on physical, sexual, and emotional abuse were not included to minimize respondent burden in the context of a comprehensive instrument as well as risk of trauma-related distress in a self-administered survey without clinical support. Use of abbreviated ACE measures and household dysfunction composite scores is established in the literature [22,23].

#### Social support and social engagement.

Perceived social support was assessed using eight items from the Multidimensional Scale of Perceived Social Support (MSPSS), capturing support from family, friends, and significant others (e.g., “I can count on my friends when things go wrong”). Each item was scored on a 7-point Likert scale from “very strongly disagree” to “very strongly agree.” Participants who completed at least six items were included; scores were averaged and standardized (z-scoring).

Social group engagement was measured using a 5-point Likert scale assessing in-person or online participation in professional, affinity, or interest-based groups over the past six months. Participants reporting “frequent” or “very frequent” engagement were dichotomously coded as having high group participation.

#### Depression history and treatment experiences.

Depression history included age of first depressive symptoms, history of ever being diagnosed with a depressive disorder, age at first diagnosis, type of diagnosing provider, diagnostic subtype (e.g., major depressive disorder), and whether the diagnosis changed over time. Age of symptom onset was categorized as childhood (0–12 years), adolescence (13–17 years), or adulthood (≥18 years).

Treatment history included whether participants had ever sought or received treatment, time from symptom onset to first treatment (<1, 1–4, or 5 + years), barriers to care (e.g., cost), treatment modalities (e.g., medication), and provider type (e.g., psychiatrist).

Perceived treatment effectiveness was rated on a 5-point Likert scale and collapsed into three categories: “not at all/slightly,” “moderately,” or “very/extremely” effective. Among participants currently receiving treatment, perceived symptom control was rated on a 5-point scale and categorized as “not at all/slightly,” “moderately,” or “completely/well” controlled.

### Statistical analysis

Six analyses were performed, corresponding to the outcomes of interest: 1) probability of never receiving a diagnosis, 2) time to diagnosis, 3) probability of never seeking treatment, 4) time from symptom onset to first treatment, 5) perceived treatment effectiveness, and 6) perceived control of current symptoms. Given minimal missingness, multivariable models were limited to complete cases. Multicollinearity was assessed using generalized variance inflation factors (GVIF), with a squared GVIF <5 indicating acceptable levels [24].

To account for variability in time at risk, each participant’s exposure time was calculated as the difference between current age and age of symptom onset. This variable was incorporated into models as a log-transformed offset term (to scale prevalence ratios) or as a covariate (to adjust for exposure duration), depending on model type [25].

The choice of statistical model for each analysis was informed by the nature of the outcome variable (binary, count-based, or categorical) and distributional assumptions. Log-Poisson regression models with robust standard errors were used for the binary outcomes in Analysis 1 (probability of never receiving a diagnosis) and Analysis 3 (probability of never seeking treatment) to estimate crude and adjusted prevalence ratios (PRs, aPRs), which better approximate relative risk when the outcome is common and the data are cross-sectional. Negative binomial regression with a log link and log-transformed offset was used for Analysis 2 (time to diagnosis) to estimate crude and adjusted rate ratios (RRs, aRRs), accounting for overdispersion in the count-based outcome. Multinomial logistic regression models with a log-transformed time-at-risk covariate were used for Analysis 4 (treatment delay) and Analysis 5 (perceived treatment effectiveness) to estimate relative risk ratios (RRRs) and adjusted relative risk ratios (aRRRs) given categorical outcomes. To address data sparsity and reduce bias, Firth’s penalized likelihood multinomial regression was used to estimate RRRs and aRRRs for Analysis 6 (perceived control of current symptoms).

All analyses included bivariate and multivariate models and were conducted‌‌ in R (version 4.5.1) [26] and Positron (version 2025.10.1) [27], using the sandwich [28] and lmtest [29] packages for log-Poisson models with robust standard errors, MASS package [30] for negative binomial regression, nnet package [31] for multinomial logistic regression, and brglm2 package [32] for Firth’s penalized likelihood multinomial regression. Two-sided statistical significance was set at α=.05.

As a sensitivity analysis, all models were re-estimated after excluding participants reporting a diagnosis of bipolar disorder (type I, type II, or type unspecified).

## Results

### Characteristics of the study sample

**Fig 1:**
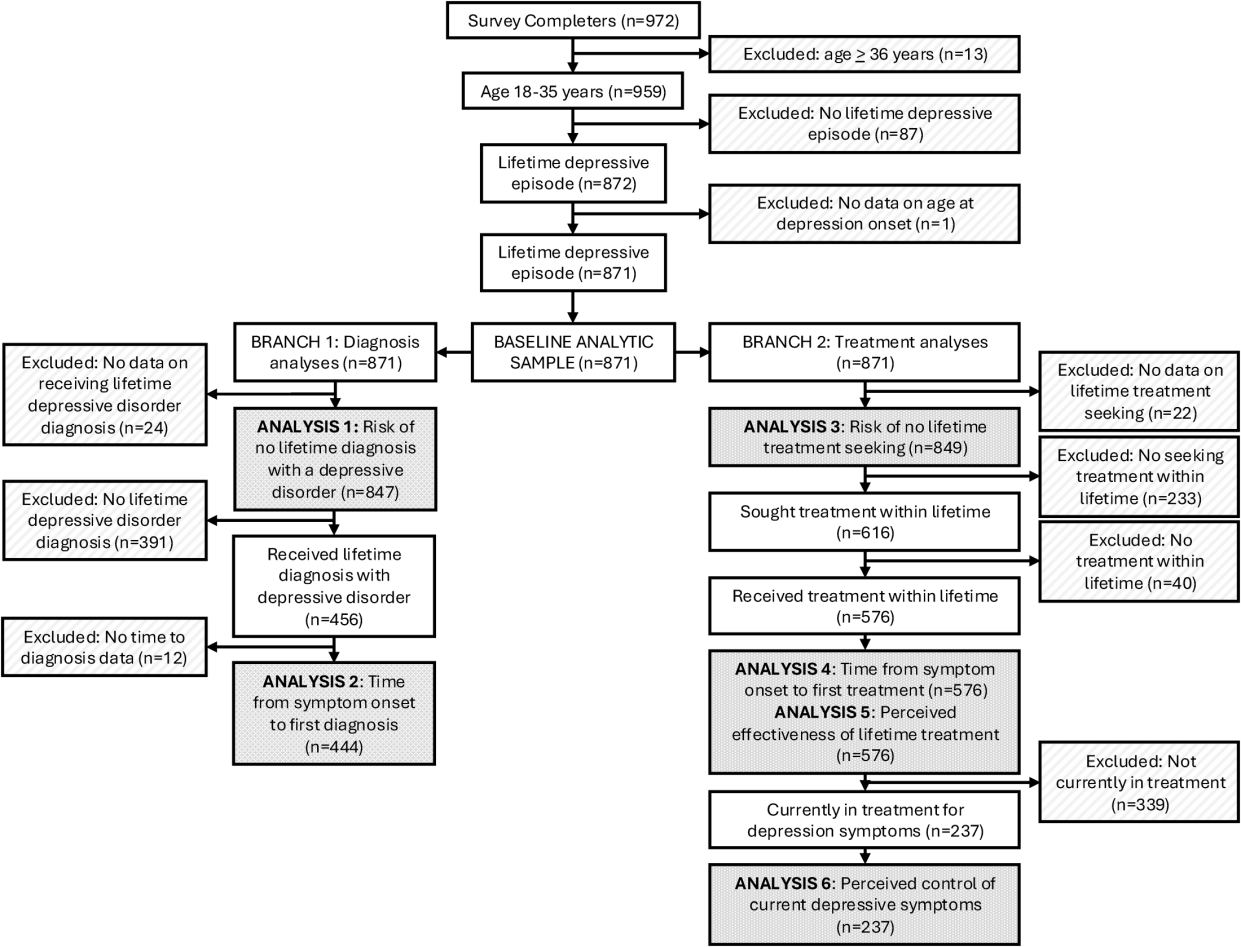
Participant flow diagram for inclusion‌‌ in analyses.

A participant flow diagram (**Fig 1**) illustrates the participant flow through screening and inclusion. Sociodemographic, clinical, and treatment characteristics are summarized in **Table 1**. Of 972 respondents who completed the survey, 871 met eligibility criteria. The median age was 29 years (IQR: 25–32). By race, 61.7% were White, 20.2% Black, 6.5% Hispanic/Latine, and 11.4% multiracial or another race. By gender, 40.2% identified as cisgender men, 47.9% as cisgender women, and 11.4% as transgender or nonbinary. By U.S. Census region, 41.8% resided in the South, 21.8% in the West, 20% in the Northeast, and 16.4% in the Midwest. Half described their community type as suburban, 37.5% as urban, and 11.9% as rural.

**Table 1:** Sample sociodemographic characteristics, mental and physical health comorbidities, and depressive disorder diagnostic and treatment histories (N = 871).

SOCIODEMOGRAPHICS	N (%)
**Age in years, median (IQR 25th-75th)**	29 (25-32)
**Age category, years**	
18-22	91 (10.4)
23-25	146 (16.8)
26-29	242 (27.8)
30-35	392 (45.0)
**Racial identity**	
African American/Black	176 (20.2)
Hispanic/Latine	57 (6.5)
Multiracial	44 (5.1)
Another racial identity	55 (6.3)
White	537 (61.7)
Missing	2 (0.2)
**Gender identity**	
Cisgender man	350 (40.2)
Cisgender woman	417 (47.9)
Nonbinary	51 (5.9)
Transfeminine	27 (3.1)
Transmasculine	21 (2.4)
Missing	5 (0.6)
**Sexual orientation**	
Heterosexual/straight	532 (61.1)
Other	335 (38.5)
Missing	4 (0.5)
**Attained a bachelor’s degree or higher**	
Yes	381 (43.7)
No	488 (56.0)
Missing	2 (0.2)
**Currently unemployed**	
Yes	111 (12.7)
No	757 (86.9)
Missing	3 (0.3)
**Self-reported community type**	
Rural	104 (11.9)
Suburban	435 (49.9)
Urban	327 (37.5)
Missing	5 (0.6)
**U.S. Census region**	
Midwest	143 (16.4)
Northeast	174 (20.0)
West	190 (21.8)
South	364 (41.8)
**PHYSICAL AND PSYCHIATRIC COMORBIDITIES**	
**Self-reported general health (item from SF-12)**	
Poor/fair	221 (25.4)
Good/very good/excellent	650 (74.6)
**Number of psychiatric comorbidities**	
2+	239 (27.4)
1	153 (17.6)
0	479 (55.0)
**PSYCHOSOCIAL FACTORS**	
**Number of adverse childhood experiences (ACEs)**	
2+	349 (40.1)
1	230 (26.4)
0	292 (33.5)
**Frequent engagement with an in-person or online social group**	
Yes	361 (41.4)
No	509 (58.4)
Missing	1 (0.1)
**Perceived social support score (subset of items from MSPSS, range: 6–40), mean (SD) (N = 855)**	29.4 (7.6)
**SYMPTOM HISTORY**	
**Timing of symptom onset**	
Childhood (0–12 years)	205 (23.5)
Adolescence (13–17 years)	331 (38.0)
Adulthood (18 + years)	335 (38.5)
**Time at risk for outcomes in years, mean (SD)**	12.0 (6.0)
**DIAGNOSTIC HISTORY**	
**Ever received a depressive disorder diagnosis (N = 847)**	
Yes	456 (53.8)
No	391 (46.2)
**Time from first symptoms to diagnosis in years, median (IQR) (N = 444)**	3 (0-7)
**Lifetime depressive disorder diagnoses (select all that apply) (N = 444)**	
Major depressive disorder or persistent depressive disorder	367 (82.7)
Bipolar disorder I or II	86 (19.4)
Mood disorder NOS or another depressive disorder	70 (15.8)
**Provider type for first diagnosis (N = 444)**	
Medical practitioner (e.g., primary care physician)	108 (24.3)
Psychiatrist	167 (37.6)
Psychologist	72 (16.2)
Therapist	69 (15.5)
Unsure	27 (6.1)
Missing	1 (0.2)
**Ever experienced a change in diagnosis (N = 444)**	
Yes, to another depressive disorder	40 (9.0)
Yes, to a non-depressive disorder	94 (21.2)
No/unsure	304 (68.5)
Missing	6 (1.4)
**TREATMENT HISTORY**	
**Ever sought treatment for depression symptoms or episodes (N = 849)**	
Yes	616 (72.6)
No	233 (27.4)
**Received treatment if they sought it (N = 616)**	
Yes	576 (93.5)
No	40 (6.5)
**Provider type for any treatment within lifetime (select all that apply) (N = 576)**	
Medical practitioner (e.g., primary care physician)	218 (37.8)
Psychiatrist	269 (46.7)
Psychologist	156 (27.1)
Therapist	255 (44.3)
**Types of treatment received (select all that apply) (N = 576)**	
Medication	382 (66.3)
Individual therapy	358 (62.2)
Family/group therapy	109 (18.9)
Intensive outpatient program or partial hospitalization	63 (10.9)
Hospitalization	91 (15.8)
**OUTCOMES**	
**Time from first symptoms to first receiving treatment (N = 576)**	
5 + years	166 (28.8)
1-4 years	181 (31.4)
Less than one year	229 (39.8)
**Perceived effectiveness of lifetime treatment (N = 576)**	
Extremely/very effective	161 (28.0)
Moderately effective	225 (39.1)
Slightly/not at all effective	190 (33.0)
**Perceived control of current symptoms (N = 237)**	
Completely/well controlled	82 (34.6)
Moderately controlled	96 (40.5)
Slightly/not at all controlled	59 (24.9)

*Note.* Denominator is the baseline analytic sample (N = 871) unless specified in the variable description. SF-12 = 12-Item Short Form Health Survey. MSPSS = Multidimensional Scale of Perceived Social Support; participants needed to answer at least 6 of 8 items to have a valid MSPSS summed score. NOS = not otherwise specified.

Most respondents (66.5%) reported at least one ACE, and 45.0% reported one or more psychiatric comorbidities. One quarter (25.4%) rated their general health as “poor” or “fair.” The mean perceived social support score (MSPSS subset, possible range = 6–40; higher scores = greater support) was 29.4 (SD: 7.6).

Self-reported depressive symptoms began in childhood for 23.5% of participants, adolescence for 38.0%, and adulthood for 38.5%. Only half (53.8%) of those with a lifetime depressive episode ever received a diagnosis. Of these, 82.7% were diagnosed with major depressive disorder or persistent depressive disorder, 19.4% with bipolar disorder type I or II, and 15.8% with a mood disorder not otherwise specified or other depressive disorder. The median time from symptom onset to diagnosis was 3 years (IQR: 0–7). The most common diagnosing provider type was a psychiatrist (37.6%), followed by primary care physician or other medical provider (24.3%), psychologist (16.2%), and therapist (15.5%); 6.1% of respondents were unsure of the provider type.

Among respondents with self-reported depressive symptoms, 27.4% never sought treatment. Of those who sought treatment, 93.5% received it; however, 31.4% experienced a delay of 1–4 years and 28.8% a delay of ≥5 years between symptom onset and first treatment. Perceived treatment effectiveness was mixed: 28.0% rated treatment as very/extremely effective, 39.1% as moderately effective, and 33.0% as slightly or not at all effective. Among those currently in treatment, 34.6% described symptoms as well/completely controlled, 40.5% as moderately controlled, and 24.9% as slightly or not at all controlled.

### Risk of never receiving diagnosis

On multivariable analysis, living in a suburban area was associated with higher probability of never receiving a diagnosis (aPR = 1.25, 95% CI: 1.04–1.49; *P =* .017) (**Table 2**). Lower probability of non-diagnosis was observed among participants with symptom onset in childhood (aPR = 0.30, 95% CI: 0.23–0.38; *P <* .001) or adolescence (aPR = 0.49, 95% CI: 0.41–0.59; *P <* .001), those identifying as nonbinary (aPR = 0.39, 95% CI: 0.20–0.75; *P =* .005), those reporting poor/fair health (aPR = 0.70, 95% CI: 0.56–0.86; *P <* .001), and those with psychiatric comorbidities (1 comorbidity: aPR = 0.65, 95% CI: 0.52–0.82; *P <* .001; ≥ 2 comorbidities: aPR = 0.30, 95% CI: 0.22–0.42, *P <* .001). Greater social support was associated with lower probability of non-diagnosis (aPR = 0.90, 95% CI: 0.83–0.98, *P =* .018).

**Table 2:** Risk of never receiving a depressive disorder diagnosis (N = 847).

	Bivariate models	Multivariable model (N = 816)
SOCIODEMOGRAPHICS	PR (95% CI), p-value	aPR (95% CI), p-value
**Age at first depression symptom onset**		
Childhood (0–12 years)	**0.24 (0.19–0.31), *P <* .001**	**0.30 (0.23–0.38), *P <* .001**
Adolescence (13–17 years)	**0.45 (0.38–0.54), *P <* .001**	**0.49 (0.41–0.59), *P <* .001**
Adulthood (18 + years)	Ref	Ref
**Gender identity**		
Cisgender man	**1.35 (1.14–1.60), *P <* .001**	0.98 (0.83–1.16), *P =* .850
Transfeminine	0.75 (0.41–1.38), *P =* .357	0.76 (0.36–1.61), *P =* .478
Transmasculine	0.56 (0.23–1.35), *P =* .199	0.58 (0.25–1.31), *P =* .191
Nonbinary	**0.37 (0.19–0.71), *P =* .003**	**0.39 (0.20–0.75), *P =* .005**
Cisgender woman	Ref	Ref
**Racial identity**		
Black/African American	**1.36 (1.11–1.67), *P =* .003**	0.98 (0.80–1.20), *P =* .879
Hispanic/Latine	0.97 (0.66–1.43), *P =* .881	0.90 (0.62–1.30), *P =* .561
Multiracial	0.60 (0.35–1.03), *P =* .063	0.79 (0.48–1.32), *P =* .373
Another racial identity	**1.49 (1.09–2.03), *P =* .012**	1.21 (0.88–1.67), *P =* .230
White	Ref	Ref
**Sexual orientation other than heterosexual**		
Yes	**0.58 (0.48–0.71), *P <* .001**	0.95 (0.77–1.16), *P =* .600
No	Ref	Ref
**Attained bachelor’s degree or higher**		
Yes	1.12 (0.95–1.33), *P =* .187	0.85 (0.70–1.02), *P =* .078
No	Ref	Ref
**Currently unemployed**		
Yes	0.96 (0.75–1.24), *P =* .776	1.10 (0.85–1.43), *P =* .473
No	Ref	Ref
**Self-reported community type**		
Rural	0.86 (0.64–1.15), *P =* .306	1.06 (0.80–1.42), *P =* .672
Suburban	1.12 (0.93–1.36), *P =* .220	**1.25 (1.04–1.49), *P =* .017**
Urban	Ref	Ref
**U.S. Census region**		
Midwest	0.90 (0.71–1.15), *P =* .402	0.91 (0.73–1.15), *P =* .449
Northeast	1.02 (0.81–1.28), *P =* .862	1.22 (0.98–1.52), *P =* .073
West	0.90 (0.72–1.13), *P =* .381	0.88 (0.71–1.09), *P =* .253
South	Ref	Ref
**PHYSICAL AND PSYCHIATRIC COMORBIDITIES**		
**Self-reported general health (item from SF-12)**		
Poor/fair	**0.59 (0.47–0.74), *P <* .001**	**0.70 (0.56–0.86), *P <* .001**
Good/very good/excellent	Ref	Ref
**Number of psychiatric comorbidities**		
2+	**0.20 (0.15–0.28), *P <* .001**	**0.30 (0.22–0.42), *P <* .001**
1	**0.58 (0.46–0.74), *P <* .001**	**0.65 (0.52–0.82), *P <* .001**
0	Ref	Ref
**PSYCHOSOCIAL FACTORS**		
**Number of adverse childhood experiences (ACEs)**		
2+	**0.55 (0.45–0.67), *P <* .001**	0.83 (0.68–1.01), *P =* .069
1	**0.79 (0.64–0.97), *P =* .022**	0.99 (0.81–1.21), *P =* .938
0	Ref	Ref
**Frequent engagement with an in-person or online social group**		
Yes	1.07 (0.89–1.27), *P =* .481	0.99 (0.83–1.19), *P =* .944
No	Ref	Ref
**Perceived social support score, z-scored (items from MSPSS)**	1.05 (0.96–1.14), *P =* .266	**0.90 (0.83–0.98), *P =* .018**

*Note.* All models incorporate a log-transformed offset term for a participant’s number of years at risk for the outcome. Multivariable results are from a complete-case analysis. Within the analytic dataset (N = 847), variable missingness ranged from 0 to 1.9% (MSPSS score). Bolding indicates statistical significance at α =  .05. SF-12 = 12-Item Short Form Health Survey. MSPSS = Multidimensional Scale of Perceived Social Support.

### Duration of diagnostic delay

On multivariable analysis, childhood and adolescent symptom onset were associated with longer diagnostic delay as compared to adult onset (childhood: aRR = 1.72; 95% CI: 1.29–2.28; *P <* .001; adolescence: aRR = 1.41; 95% CI: 1.09–1.83; *P =* .009) (**Table 3**). Additional predictors of longer delay included Hispanic/Latine identity (aRR = 1.58; 95% CI: 1.11–2.26, *P =* .011), higher education (bachelor’s degree or higher: aRR = 1.26; 95% CI: 1.02–1.55, *P =* .034), and unemployment (aRR = 1.34; 95% CI: 1.01–1.77; *P =* .041). Factors associated with shorter delays included greater social support (aRR = 0.85; 95% CI: 0.77–0.94, *P =* .001), transfeminine gender identity (aRR = 0.56; 95% CI: 0.32–0.99, *P =* .045), and being unsure of the diagnosing provider (aRR = 0.63; 95% CI: 0.42–0.94, *P =* .025).

**Table 3:** Time in years from symptom onset to first diagnosis with a depressive disorder (N = 444).

	Bivariate models	Multivariable model (N = 429)
SOCIODEMOGRAPHICS	RR (95% CI), p-value	aRR (95% CI), p-value
**Age at first depression symptom onset**		
Childhood (0–12 years)	**1.98 (1.53–2.56), *P <* .001**	**1.72 (1.29–2.28), *P <* .001**
Adolescence (13–17 years)	**1.47 (1.14–1.89), *P =* .003**	**1.41 (1.09–1.83), *P =* .009**
Adulthood (18 + years)	1.00 (Reference)	1.00 (Reference)
**Gender identity**		
Cisgender man	0.87 (0.70–1.09), *P =* .220	0.83 (0.66–1.03), *P =* .088
Transfeminine	**0.50 (0.29–0.89), *P =* .017**	**0.56 (0.32–0.99), *P =* .045**
Transmasculine	0.98 (0.57–1.70), *P =* .954	1.07 (0.63–1.81), *P =* .793
Nonbinary	1.18 (0.85–1.65), *P =* .323	1.21 (0.87–1.69), *P =* .255
Cisgender woman	1.00 (Reference)	1.00 (Reference)
**Racial identity**		
Black/African American	0.82 (0.63–1.08), *P =* .158	1.00 (0.76–1.30), *P =* .971
Hispanic/Latine	**1.50 (1.04–2.17), *P =* .030**	**1.58 (1.11–2.26), *P =* .011**
Multiracial	1.05 (0.71–1.55), *P =* .800	0.88 (0.61–1.29), *P =* .518
Another racial identity	1.12 (0.71–1.77), *P =* .616	1.10 (0.70–1.72), *P =* .673
White	1.00 (Reference)	1.00 (Reference)
**Sexual orientation other than heterosexual**		
Yes	1.04 (0.86–1.27), *P =* .669	0.97 (0.79–1.19), *P =* .796
No	1.00 (Reference)	1.00 (Reference)
**Attained bachelor’s degree or higher**		
Yes	0.98 (0.80–1.19), *P =* .810	**1.26 (1.02–1.55), *P =* .034**
No	1.00 (Reference)	1.00 (Reference)
**Currently unemployed**		
Yes	**1.33 (1.01–1.76), *P =* .044**	**1.34 (1.01–1.77), *P =* .041**
No	1.00 (Reference)	1.00 (Reference)
**Self-reported community type**		
Rural	1.33 (0.98–1.82), *P =* .070	1.27 (0.93–1.73), *P =* .130
Suburban	1.08 (0.88–1.34), *P =* .449	0.97 (0.78–1.19), *P =* .745
Urban	1.00 (Reference)	1.00 (Reference)
**U.S. Census region**		
Midwest	1.14 (0.86–1.51), *P =* .370	1.11 (0.85–1.45), *P =* .436
Northeast	1.18 (0.91–1.54), *P =* .218	1.12 (0.86–1.46), *P =* .391
West	1.00 (0.78–1.30), *P =* .983	1.06 (0.82–1.36), *P =* .649
South	1.00 (Reference)	1.00 (Reference)
**DIAGNOSTIC HISTORY**		
**Provider type for first diagnosis**		
Medical practitioner (e.g., primary care physician)	0.94 (0.73–1.21), *P =* .634	0.94 (0.74–1.20), *P =* .645
Psychologist	0.95 (0.71–1.27), *P =* .737	1.14 (0.86–1.52), *P =* .350
Therapist	0.97 (0.72–1.30), *P =* .834	1.15 (0.86–1.53), *P =* .340
Unsure	0.78 (0.51–1.19), *P =* .250	**0.63 (0.42–0.94), *P =* .025**
Psychiatrist	1.00 (Reference)	1.00 (Reference)
**Ever experienced a change in diagnosis**		
Yes, to another depressive disorder	**0.69 (0.48–0.98), *P =* .037**	0.81 (0.57–1.14), *P =* .225
Yes, to a non-depressive disorder	0.80 (0.63–1.02), *P =* .068	0.90 (0.71–1.15), *P =* .419
No/unsure	1.00 (Reference)	1.00 (Reference)
**PHYSICAL AND PSYCHIATRIC COMORBIDITIES**		
**Self-reported general health (item from SF-12)**		
Poor/fair	1.03 (0.84–1.27), *P =* .777	0.94 (0.77–1.15), *P =* .554
Good/very good/excellent	1.00 (Reference)	1.00 (Reference)
**Number of psychiatric comorbidities**		
2+	1.04 (0.83–1.29), *P =* .738	0.88 (0.70–1.11), *P =* .294
1	1.03 (0.78–1.36), *P =* .839	0.89 (0.68–1.16), *P =* .383
0	1.00 (Reference)	1.00 (Reference)
**PSYCHOSOCIAL FACTORS**		
**Number of adverse childhood experiences (ACEs)**		
2+	1.17 (0.93–1.48), *P =* .184	1.06 (0.83–1.36), *P =* .617
1	1.12 (0.86–1.48), *P =* .401	1.02 (0.78–1.33), *P =* .904
0	1.00 (Reference)	1.00 (Reference)
**Frequent engagement with an in-person or online social group**		
Yes	**0.77 (0.63–0.94), *P =* .009**	0.87 (0.70–1.07), *P =* .175
No	1.00 (Reference)	1.00 (Reference)
**Perceived social support score, z-scored (items from MSPSS)**	**0.82 (0.75–0.90), *P <* .001**	**0.85 (0.77–0.94), *P =* .001**

*Note.* All models incorporate a log-transformed offset term for a participant’s number of years at risk for the outcome. Multivariable results are from a complete-case analysis. Within the analytic dataset (N = 444), variable missingness ranged from 0 to 1.4% (ever experienced a change in diagnosis). Bolding indicates statistical significance at α =  .05. SF-12 = 12-Item Short Form Health Survey. MSPSS = Multidimensional Scale of Perceived Social Support.

### Risk of not seeking treatment

Being unemployed (aPR = 1.42, 95% CI: 1.02–1.98, *P =* .039) and living in a suburban setting (aPR = 1.45, 95% CI: 1.11–1.89, *P =* .006) were associated with a higher probability of never seeking treatment for depressive symptoms (**Table 4**). Conversely, a lower probability was observed among participants with childhood (aPR = 0.27, 95% CI: 0.19–0.39, *P <* .001) or adolescent (aPR = 0.50, 95% CI: 0.38–0.64, *P <* .001) symptom onset, those with psychiatric comorbidities (1 comorbidity: aPR = 0.39, 95% CI: 0.25–0.60, *P <* .001; ≥ 2 comorbidities: aPR = 0.15, 95% CI: 0.09–0.28, *P <* .001), and those with greater social support (aPR = 0.85, 95% CI: 0.75–0.96, *P =* .009).

**Table 4:** Risk of not seeking treatment for depressive symptoms in lifetime (N = 849).

	Bivariate models	Multivariable model (N = 804)
SOCIODEMOGRAPHICS	PR (95% CI), p-value	aPR (95% CI), p-value
**Age at first depression symptom onset**		
Childhood (0–12 years)	**0.21 (0.15–0.31), *P <* .001**	**0.27 (0.19–0.39), *P <* .001**
Adolescence (13–17 years)	**0.49 (0.38–0.63), *P <* .001**	**0.50 (0.38–0.64), *P <* .001**
Adulthood (18 + years)	1.00 (Reference)	1.00 (Reference)
**Gender identity**		
Cisgender man	**1.70 (1.32–2.18), *P <* .001**	1.12 (0.87–1.45), *P =* .376
Transfeminine	0.55 (0.18–1.72), *P =* .304	0.57 (0.14–2.36), *P =* .435
Transmasculine	0.93 (0.34–2.52), *P =* .879	1.01 (0.39–2.64), *P =* .980
Nonbinary	**0.39 (0.16–0.97), *P =* .042**	0.48 (0.19–1.20), *P =* .115
Cisgender woman	1.00 (Reference)	1.00 (Reference)
**Racial identity**		
Black/African American	**1.71 (1.28–2.28), *P <* .001**	1.24 (0.94–1.63), *P =* .131
Hispanic/Latine	1.51 (0.96–2.39), *P =* .076	1.40 (0.88–2.24), *P =* .152
Multiracial	0.54 (0.24–1.20), *P =* .131	0.77 (0.37–1.61), *P =* .489
Another racial identity	**1.96 (1.32–2.92), *P <* .001**	1.45 (0.99–2.13), *P =* .059
White	1.00 (Reference)	1.00 (Reference)
**Sexual orientation other than heterosexual**		
Yes	**0.49 (0.37–0.65), *P <* .001**	0.82 (0.60–1.11), *P =* .194
No	1.00 (Reference)	1.00 (Reference)
**Attained bachelor’s degree or higher**		
Yes	0.90 (0.71–1.14), *P =* .379	0.79 (0.61–1.04), *P =* .089
No	1.00 (Reference)	1.00 (Reference)
**Currently unemployed**		
Yes	1.30 (0.95–1.78), *P =* .101	**1.42 (1.02–1.98), *P =* .039**
No	1.00 (Reference)	1.00 (Reference)
**Self-reported community type**		
Rural	0.95 (0.62–1.46), *P =* .821	1.17 (0.78–1.77), *P =* .443
Suburban	**1.36 (1.04–1.79), *P =* .027**	**1.45 (1.11–1.89), *P =* .006**
Urban	1.00 (Reference)	1.00 (Reference)
**U.S. Census region**		
Midwest	1.04 (0.75–1.45), *P =* .814	1.15 (0.84–1.56), *P =* .392
Northeast	0.84 (0.60–1.19), *P =* .329	0.99 (0.71–1.39), *P =* .961
West	0.93 (0.68–1.28), *P =* .659	0.87 (0.65–1.17), *P =* .350
South	1.00 (Reference)	1.00 (Reference)
**PHYSICAL AND PSYCHIATRIC COMORBIDITIES**		
**Self-reported general health (item from SF-12)**		
Poor/fair	**0.66 (0.49–0.90), *P =* .008**	0.74 (0.54–1.01), *P =* .054
Good/very good/excellent	1.00 (Reference)	1.00 (Reference)
**Number of psychiatric comorbidities**		
2+	**0.09 (0.05–0.16), *P <* .001**	**0.15 (0.09–0.28), *P <* .001**
1	**0.34 (0.22–0.51), *P <* .001**	**0.39 (0.25–0.60), *P <* .001**
0	1.00 (Reference)	1.00 (Reference)
**PSYCHOSOCIAL FACTORS**		
**Number of adverse childhood experiences (ACEs)**		
2+	**0.49 (0.37–0.65), *P <* .001**	0.76 (0.57–1.01), *P =* .060
1	0.76 (0.57–1.01), *P =* .060	1.01 (0.75–1.35), *P =* .959
0	1.00 (Reference)	1.00 (Reference)
**Frequent engagement with an in-person or online social group**		
Yes	0.98 (0.76–1.26), *P =* .881	1.02 (0.79–1.32), *P =* .883
No	1.00 (Reference)	1.00 (Reference)
**Perceived social support score, z-scored (items from MSPSS)**	0.94 (0.84–1.06), *P =* .331	**0.85 (0.75–0.96), *P =* .009**

*Note.* All models incorporate a log-transformed offset term for a participant’s number of years at risk for the outcome. Multivariable results are from a complete-case analysis. Within the analytic dataset (N = 849), variable missingness ranged from 0 to 1.9% (MSPSS score). Bolding indicates statistical significance at α =  .05. SF-12 = 12-Item Short Form Health Survey. MSPSS = Multidimensional Scale of Perceived Social Support.

### Duration of treatment delay

Duration of treatment delay varied by age of onset (**Table 5**). As compared to adult onset, childhood onset was associated with a > 4-fold higher probability of delay ≥ 5 years (aRRR = 4.23; 95% CI: 1.95–9.20, *P <* .001) and adolescent onset with nearly twice the probability of a 1–4-year delay (aRRR = 1.84; 95% CI: 1.07–3.16, *P =* .028). Participants with ACEs were more likely to experience treatment delays of 1−4 years (1 ACE: aRRR = 2.02, 95% CI: 1.12–3.67, *P =* .020; ≥ 2 ACEs: aRRR = 1.76, 95% CI: 1.01–3.06, *P =* .047). Greater social support was associated with a lower probability of ≥5-year delay (aRRR = 0.70, 95% CI: 0.54–0.91, *P =* .009).

**Table 5:** Time in years from symptom onset to first receiving treatment for depressive symptoms (N = 576).

	Bivariate models		Multivariable model (N = 556)	
	1-4 years vs. <1 year	5 + years vs. <1 year	1-4 years vs. <1 year	5 + years vs. <1 year
SOCIODEMOGRAPHICS	RRR (95% CI), p-value	RRR (95% CI), p-value	aRRR (95% CI), p-value	aRRR (95% CI), p-value
**Age at first depression symptom onset**				
Childhood (0–12 years)	**2.55 (1.39–4.67), *P =* .002**	**4.66 (2.32–9.33), *P <* .001**	1.73 (0.87–3.47), *P =* .120	**4.23 (1.95–9.20), *P <* .001**
Adolescence (13–17 years)	**2.37 (1.46–3.86), *P <* .001**	**2.03 (1.12–3.68), *P =* .020**	**1.84 (1.07–3.16), *P =* .028**	1.78 (0.93–3.38), *P =* .080
Adulthood (18 + years)	1.00 (Reference)	1.00 (Reference)	1.00 (Reference)	1.00 (Reference)
**Gender identity**				
Cisgender man	0.85 (0.55–1.31), *P =* .459	0.81 (0.51–1.28), *P =* .365	0.88 (0.54–1.42), *P =* .599	0.85 (0.51–1.42), *P =* .532
Transfeminine	2.60 (0.91–7.43), *P =* .074	0.83 (0.19–3.59), *P =* .808	2.54 (0.82–7.81), *P =* .105	1.17 (0.26–5.30), *P =* .842
Transmasculine	0.77 (0.22–2.71), *P =* .684	1.00 (0.29–3.41), *P =* .998	0.61 (0.16–2.39), *P =* .479	1.12 (0.28–4.53), *P =* .871
Nonbinary	**3.57 (1.43–8.89), *P =* .006**	**2.73 (1.07–6.99), *P =* .036**	2.26 (0.84–6.05), *P =* .106	2.69 (0.97–7.50), *P =* .057
Cisgender woman	1.00 (Reference)	1.00 (Reference)	1.00 (Reference)	1.00 (Reference)
**Racial identity**				
Black/African American	0.87 (0.52–1.46), *P =* .605	0.88 (0.49–1.58), *P =* .660	1.10 (0.62–1.95), *P =* .745	0.99 (0.52–1.87), *P =* .979
Hispanic/Latine	0.51 (0.20–1.29), *P =* .154	0.60 (0.25–1.46), *P =* .262	0.45 (0.16–1.21), *P =* .114	0.45 (0.17–1.22), *P =* .119
Multiracial	1.26 (0.55–2.87), *P =* .582	0.85 (0.34–2.13), *P =* .722	0.89 (0.36–2.20), *P =* .807	0.49 (0.18–1.36), *P =* .174
Another racial identity	0.48 (0.15–1.57), *P =* .223	1.64 (0.64–4.20), *P =* .307	0.45 (0.13–1.59), *P =* .215	1.28 (0.45–3.61), *P =* .641
White	1.00 (Reference)	1.00 (Reference)	1.00 (Reference)	1.00 (Reference)
**Sexual orientation other than heterosexual**				
Yes	**1.70 (1.14–2.55), *P =* .010**	1.35 (0.88–2.06), *P =* .171	1.19 (0.75–1.89), *P =* .458	1.04 (0.63–1.72), *P =* .873
No	1.00 (Reference)	1.00 (Reference)	1.00 (Reference)	1.00 (Reference)
**Attained bachelor’s degree or higher**				
Yes	**0.59 (0.39–0.89), *P =* .012**	0.82 (0.53–1.27), *P =* .375	0.80 (0.49–1.31), *P =* .380	1.25 (0.74–2.11), *P =* .398
No	1.00 (Reference)	1.00 (Reference)	1.00 (Reference)	1.00 (Reference)
**Currently unemployed**				
Yes	**2.23 (1.15–4.30), *P =* .017**	1.73 (0.86–3.48), *P =* .127	1.80 (0.86–3.76), *P =* .116	1.42 (0.64–3.12), *P =* .388
No	1.00 (Reference)	1.00 (Reference)	1.00 (Reference)	1.00 (Reference)
**Self-reported community type**				
Rural	1.42 (0.74–2.75), *P =* .291	1.36 (0.67–2.72), *P =* .394	1.16 (0.55–2.43), *P =* .702	1.25 (0.57–2.77), *P =* .576
Suburban	0.97 (0.63–1.48), *P =* .880	1.13 (0.72–1.79), *P =* .591	0.84 (0.52–1.36), *P =* .480	1.03 (0.62–1.71), *P =* .902
Urban	1.00 (Reference)	1.00 (Reference)	1.00 (Reference)	1.00 (Reference)
**U.S. Census region**				
Midwest	1.27 (0.70–2.30), *P =* .435	1.19 (0.63–2.23), *P =* .597	1.34 (0.70–2.57), *P =* .372	1.07 (0.53–2.14), *P =* .852
Northeast	1.00 (0.58–1.71), *P =* .995	1.18 (0.67–2.07), *P =* .561	1.16 (0.64–2.10), *P =* .616	1.20 (0.64–2.23), *P =* .574
West	0.89 (0.53–1.51), *P =* .672	0.96 (0.55–1.68), *P =* .886	1.18 (0.66–2.10), *P =* .585	1.08 (0.58–1.99), *P =* .817
South	1.00 (Reference)	1.00 (Reference)	1.00 (Reference)	1.00 (Reference)
**TREATMENT HISTORY**				
**Provider type for any treatment within lifetime (check-all-that-apply)**				
**Medical practitioner (e.g., primary care physician)**				
Yes	1.28 (0.85–1.93), *P =* .243	0.91 (0.58–1.41), *P =* .668	0.99 (0.62–1.58), *P =* .965	0.66 (0.39–1.09), *P =* .103
No	1.00 (Reference)	1.00 (Reference)	1.00 (Reference)	1.00 (Reference)
**Psychiatrist**				
Yes	**1.55 (1.03–2.32), *P =* .034**	1.09 (0.71–1.69), *P =* .682	1.19 (0.73–1.93), *P =* .483	0.93 (0.55–1.56), *P =* .773
No	1.00 (Reference)	1.00 (Reference)	1.00 (Reference)	1.00 (Reference)
**Psychologist**				
Yes	0.99 (0.64–1.55), *P =* .974	0.78 (0.48–1.25), *P =* .304	0.78 (0.47–1.29), *P =* .331	0.67 (0.39–1.16), *P =* .156
No	1.00 (Reference)	1.00 (Reference)	1.00 (Reference)	1.00 (Reference)
**Therapist**				
Yes	**1.59 (1.06–2.38), *P =* .024**	1.28 (0.84–1.97), *P =* .256	1.38 (0.85–2.22), *P =* .189	1.29 (0.77–2.17), *P =* .325
No	1.00 (Reference)	1.00 (Reference)	1.00 (Reference)	1.00 (Reference)
**PHYSICAL AND PSYCHIATRIC COMORBIDITIES**				
**Self-reported general health (item from SF-12)**				
Poor/fair	1.30 (0.83–2.04), *P =* .255	1.20 (0.75–1.92), *P =* .456	0.99 (0.60–1.63), *P =* .961	0.97 (0.57–1.64), *P =* .897
Good/very good/excellent	1.00 (Reference)	1.00 (Reference)	1.00 (Reference)	1.00 (Reference)
**Number of psychiatric comorbidities**				
2+	1.59 (1.00–2.53), *P =* .050	1.35 (0.83–2.19), *P =* .230	0.85 (0.49–1.47), *P =* .555	1.04 (0.58–1.85), *P =* .894
1	1.65 (0.98–2.78), *P =* .059	1.00 (0.56–1.81), *P =* .994	1.26 (0.72–2.22), *P =* .416	0.88 (0.46–1.65), *P =* .683
0	1.00 (Reference)	1.00 (Reference)	1.00 (Reference)	1.00 (Reference)
**PSYCHOSOCIAL FACTORS**				
**Number of adverse childhood experiences (ACEs)**				
2+	**2.16 (1.30–3.57), *P =* .003**	1.02 (0.62–1.69), *P =* .932	**1.76 (1.01–3.06), *P =* .047**	0.83 (0.47–1.47), *P =* .522
1	**2.28 (1.31–3.95), *P =* .003**	0.79 (0.44–1.43), *P =* .445	**2.02 (1.12–3.67), *P =* .020**	0.64 (0.34–1.23), *P =* .179
0	1.00 (Reference)	1.00 (Reference)	1.00 (Reference)	1.00 (Reference)
**Frequent engagement with an in-person or online social group**				
Yes	0.71 (0.47–1.07), *P =* .101	0.77 (0.50–1.20), *P =* .250	0.92 (0.56–1.51), *P =* .750	0.95 (0.57–1.58), *P =* .837
No	1.00 (Reference)	1.00 (Reference)	1.00 (Reference)	1.00 (Reference)
**Perceived social support score, z-scored (items from MSPSS)**	**0.72 (0.58–0.89), *P =* .003**	**0.69 (0.55–0.87), *P =* .002**	0.83 (0.64–1.07), *P =* .143	**0.70 (0.54–0.91), *P =* .009**

*Note.* All models incorporate a log-transformed offset term for a participant’s number of years at risk for the outcome. Multivariable results are from a complete-case analysis. Within the analytic dataset (N = 576), variable missingness ranged from 0 to 1.9% (MSPSS score). Bolding indicates statistical significance at α =  .05. SF-12 = 12-Item Short Form Health Survey. MSPSS = Multidimensional Scale of Perceived Social Support.

### Perceived treatment effectiveness

As compared to adult onset, childhood and adolescent symptom onset were both associated with lower probability of perceiving treatment as moderately effective (childhood: aRRR = 0.27, 95% CI: 0.12–0.59, *P =* .001; adolescence: aRRR = 0.29, 95% CI: 0.15–0.56, *P <* .001) or very/extremely effective (childhood: aRRR = 0.25, 95% CI: 0.10–0.62, *P =* .003; adolescence: aRRR = 0.29, 95% CI: 0.14–0.61, *P =* .001) (**S2 Appendix**). Lower perceived effectiveness was also reported by respondents with poor/fair general health (aRRR = 0.38, 95% CI: 0.19–0.74, *P =* .005) or a sexual orientation other than heterosexual (aRRR = 0.50, 95% CI: 0.28–0.91, *P =* .023).

Higher perceived effectiveness was associated with frequent in-person or online group engagement (aRRR = 3.80, 95% CI: 2.05–7.03, *P <* .001), higher perceived social support (aRRR = 2.56, 95% CI: 1.80–3.64, *P <* .001), and having a therapist as a treatment provider (aRRR = 2.11, 95% CI: 1.00–4.41, *P =* .049).

### Perceived current control of symptoms

Respondents reporting poor/fair general health (aRRR = 0.19, 95% CI: 0.08–0.49, *P <* .001) and those residing in the Midwest versus the South (aRRR = 0.19, 95% CI: 0.04–0.79, *P =* .023) had a lower probability of rating symptoms as well/completely controlled (**S3 Appendix**). Greater social support was associated with a higher probability of well-controlled symptoms (aRRR = 2.36, 95% CI: 1.39–3.98, *P =* .001). A higher probability of moderate control was observed among suburban versus urban participants (aRRR = 2.64, 95% CI: 1.10–6.36, *P =* .030) and participants with a history of psychiatric hospitalization or inpatient treatment (aRRR = 3.82, 95% CI: 1.15–12.69, *P =* .029).

### Sensitivity analysis

Sensitivity analyses excluding participants with any reported history of bipolar disorder diagnosis (N = 94 excluded) yielded results consistent with the primary analyses. Forest plots comparing effect estimates from the full and restricted samples are presented in S4 Appendix.

## Discussion

To our knowledge, this is the first national cross-sectional study to examine diagnostic and treatment delays for depressive disorders specifically among U.S. young adults. The finding that such delays are highly prevalent has important implications. First, the results suggest depressive disorders may be even more common among young adults than previously estimated. Nearly half of respondents with a self-reported lifetime depressive episode never received a formal diagnosis, and among those who did, the median delay from symptom onset to diagnosis was several years. Furthermore, over one-quarter of respondents never sought treatment. These findings reinforce emerging evidence that treatment among young adults is not keeping pace with the growing prevalence of depressive disorders [4]. Elucidating key contributors to these diagnostic and treatment challenges – and factors that may mitigate them – is critical to addressing the mental health crisis affecting U.S. young adults.

Second, this study identified predictors of diagnostic and treatment delay. Symptom onset in childhood or adolescence was associated with longer time to diagnosis, greater probability of prolonged treatment delay, and lower perceived treatment effectiveness. These findings are consistent with prior research linking early-onset depression to worse long-term outcomes. They further suggest that trajectories of mental health care may differ fundamentally for individuals whose symptoms begin in youth versus adulthood. Prospective research is needed to characterize these longitudinal trajectories – spanning symptom onset, diagnosis, treatment initiation, recovery, and recurrence —to identify intervention targets [33].

Third, greater social support was associated with shorter time to diagnosis; lower probability of non-diagnosis, non-treatment, and prolonged treatment delay; and higher perceived treatment effectiveness. Prior studies have shown that social support protects against depression onset and mitigates poor outcomes such as suicidal behavior [8–12,34,35]. However, its role in shaping trajectories from symptom onset to receipt of care has been less clear. The present findings begin to fill this knowledge gap, suggesting that social support is associated with timelier access to diagnosis and treatment as well as more positive treatment experiences. Frequent participation in social groups was also associated with higher perceived treatment effectiveness. Importantly, these associations may be bidirectional: social support could facilitate help-seeking and engagement with care, but individuals who receive timely diagnosis and treatment may also be better positioned to maintain social functioning and supportive relationships. Since the cross-sectional design of the study precludes determination of causal mechanism, social support should be interpreted as a correlate. Future research is needed to determine whether interventions promoting connection and belonging among young people – whether through peer support, mentorship, or community engagement – can improve help-seeking behaviors and outcomes.

This study has limitations. Use of self-reported data introduces the potential for recall bias; participants were asked to recall age at first symptoms, age at diagnosis, and age at first treatment, among other variables. This could also introduce information bias, wherein participants with early-onset symptoms were required to recall events from a more distant past as compared to participants with adult-onset symptoms, potentially leading to differential misclassification of delay duration across onset groups. Notwithstanding these limitations, self-reported data likely provide a more complete picture of diagnostic and treatment delays than studies using administrative or electronic health record data. Individuals who have never engaged with the health care system may be “invisible” in clinical datasets. Because symptom onset typically precedes health care contact, affected individuals are also arguably best positioned to estimate the duration of delays they experience.

Use of an online survey platform may limit generalizability, as Prolific participants could be more technologically literate or more willing to engage in research than the broader population. Specifically, recruitment through Prolific could introduce selection bias toward individuals who are healthy enough to seek out and participate in online surveys, are attuned to their own symptoms, or are comfortable with self-disclosure online of sensitive psychiatric history. Compensation ($3 for survey completion) could also differentially attract participants based on socioeconomic factors. These results therefore should not be interpreted as representative national epidemiological estimates of diagnostic or treatment delay prevalence without appropriate weighting or benchmarking against population-based data. However, Internet and smartphone use are nearly universal among 18–35-year-olds [36], and the study achieved a geographically and demographically diverse national sample, enhancing external validity.

The study sample included participants who reported ultimately receiving a diagnosis of bipolar disorder, which may differ from unipolar depressive disorders in onset pattern, diagnostic trajectory, and treatment approach. Many individuals with bipolar disorder initially present with depressive episodes and may be diagnosed with and treated for a unipolar depressive disorder before ultimately receiving a bipolar diagnosis. Their experiences are therefore relevant to understanding diagnostic delays for mood disorders broadly. However, we acknowledge that combining unipolar and bipolar cases may obscure condition-specific heterogeneity in delay estimates.

Finally, the cross-sectional design of the study precludes causal inference. For example, the association between social support and timelier diagnosis and treatment may indicate that supportive networks help individuals access care – or that those predisposed to seek care are also more likely to maintain supportive relationships. Nonetheless, the strength and consistency of these associations highlight the importance of further research to clarify causal mechanisms. Randomized controlled trials could help to determine whether interventions that enhance social support improve the timeliness and effectiveness of mental health care. Prospective longitudinal studies are also needed to map care trajectories among individuals with early-onset depressive symptoms, who appear to be at greatest risk for delayed diagnosis and treatment.

## Conclusion

In this national cross-sectional study, diagnostic and treatment delays for depressive disorders were common among U.S. young adults. The median diagnostic delay was 3 years, and more than 60% of respondents reported treatment delays exceeding 1 year. Early symptom onset in childhood or adolescence was associated with greater likelihood of delayed diagnosis and treatment, whereas higher levels of social support were associated with shorter delays. These findings highlight the need for further study of mental health care trajectories among youth and young adults. Longitudinal and intervention-based research is needed to determine whether strengthening social support can reduce delays and improve outcomes in this vulnerable population.

## Supporting information

S1 Appendix STROBE checklist. (DOCX)

S2 Appendix Perceived effectiveness of lifetime treatment for depressive symptoms. (DOCX)

S3 Appendix Perceived control of current depressive symptoms. (DOCX)

S4 Appendix Sensitivity analysis excluding participants with bipolar disorder diagnosis. (DOCX)
