# Supplementary material for: Delays in diagnosis and treatment of depressive disorder among young adults: A national online survey-based cross-sectional study
Source: PLoS One. 2026 Jun 12;21(6):e0351402. doi: 10.1371/journal.pone.0351402 (PMC13262879; doi:10.1371/journal.pone.0351402)
Supplement: S1 Appendix — (DOCX) [file pone.0351402.s001.docx]

# S1 Appendix. STROBE Checklist for Cross-Sectional Studies

*Delays in diagnosis and treatment of depressive disorder among young adults: A national online survey-based cross-sectional study*

| **Item No** | **Section/Topic** | **Recommendation** | **Page** |
| --- | --- | --- | --- |
| 1a | **Title and abstract** | Indicate the study’s design with a commonly used term in the title or the abstract | 1-2 |
| 1b | **Title and abstract** | Provide in the abstract an informative and balanced summary of what was done and what was found | 2 |
| 2 | **Background/rationale** | Explain the scientific background and rationale for the investigation being reported | 4-5 |
| 3 | **Objectives** | State specific objectives, including any prespecified hypotheses | 4-5 |
| 4 | **Study design** | Present key elements of study design early in the paper | 5 |
| 5 | **Setting** | Describe the setting, locations, and relevant dates, including periods of recruitment, exposure, follow-up, and data collection | 5 |
| 6a | **Participants** | Give the eligibility criteria, and the sources and methods of selection of participants | 5 |
| 7 | **Variables** | Clearly define all outcomes, exposures, predictors, potential confounders, and effect modifiers. Give diagnostic criteria, if applicable | 6-8 |
| 8* | **Data sources/measurement** | For each variable of interest, give sources of data and details of methods of assessment (measurement). Describe comparability of assessment methods if there is more than one group | 6-8 |
| 9 | **Bias** | Describe any efforts to address potential sources of bias | 9-10, 34-36 |
| 10 | **Study size** | Explain how the study size was arrived at | 5 |
| 11 | **Quantitative variables** | Explain how quantitative variables were handled in the analyses. If applicable, describe which groupings were chosen and why | 6-10 |
| 12a | **Statistical methods** | Describe all statistical methods, including those used to control for confounding | 9-10 |
| 12b | **Statistical methods** | Describe any methods used to examine subgroups and interactions | 9-10 |
| 12c | **Statistical methods** | Explain how missing data were addressed | 9-10 |
| 12d | **Statistical methods** | If applicable, describe analytical methods taking account of sampling strategy | 9-10 |
| 12e | **Statistical methods** | Describe any sensitivity analyses | 10 |
| 13a* | **Participants** | Report numbers of individuals at each stage of study—eg numbers potentially eligible, examined for eligibility, confirmed eligible, included in the study, completing follow-up, and analysed | 10-11 |
| 13b* | **Participants** | Give reasons for non-participation at each stage | Figure 1 |
| 13c* | **Participants** | Consider use of a flow diagram | Figure 1 |
| 14a* | **Descriptive data** | Give characteristics of study participants (eg demographic, clinical, social) and information on exposures and potential confounders | 10-15 |
| 14b* | **Descriptive data** | Indicate number of participants with missing data for each variable of interest | 12-15 |
| 15* | **Outcome data** | Report numbers of outcome events or summary measures | 15-32 |
| 16a | **Main results** | Give unadjusted estimates and, if applicable, confounder-adjusted estimates and their precision (eg, 95% confidence interval). Make clear which confounders were adjusted for and why they were included | 15-32 |
| 16b | **Main results** | Report category boundaries when continuous variables were categorized | 15-32 |
| 16c | **Main results** | If relevant, consider translating estimates of relative risk into absolute risk for a meaningful time period | N/A |
| 17 | **Other analyses** | Report other analyses done—eg analyses of subgroups and interactions, and sensitivity analyses | 33, S4 Appendix |
| 18 | **Key results** | Summarise key results with reference to study objectives | 33-34 |
| 19 | **Limitations** | Discuss limitations of the study, taking into account sources of potential bias or imprecision. Discuss both direction and magnitude of any potential bias | 34-36 |
| 20 | **Interpretation** | Give a cautious overall interpretation of results considering objectives, limitations, multiplicity of analyses, results from similar studies, and other relevant evidence | 33-34, 36 |
| 21 | **Generalisability** | Discuss the generalisability (external validity) of the study results | 35 |
| 22 | **Funding** | Give the source of funding and the role of the funders for the present study and, if applicable, for the original study on which the present article is based | Funding Statement |
