# Supplementary material for: Delays in diagnosis and treatment of depressive disorder among young adults: A national online survey-based cross-sectional study
Source: PLoS One. 2026 Jun 12;21(6):e0351402. doi: 10.1371/journal.pone.0351402 (PMC13262879; doi:10.1371/journal.pone.0351402)
Supplement: S2 Appendix — (DOCX) [file pone.0351402.s002.docx]

**S2 Appendix**. Perceived effectiveness of lifetime treatment for depressive symptoms (N=576)

|  | **Bivariate models** | | **Multivariable model (N=556)** | |
| --- | --- | --- | --- | --- |
|  | **Moderately effective vs slightly/not at all effective** | **Extremely/very effective vs slightly/not at all effective** | **Moderately effective vs slightly/not at all effective** | **Extremely/very effective vs slightly/not at all effective** |
| **SOCIODEMOGRAPHICS** | **RRR (95% CI),**  **p-value** | **RRR (95% CI),**  **p-value** | **aRRR (95% CI),**  **p-value** | **aRRR (95% CI),**  **p-value** |
| **Age at first depression symptom onset** |  |  |  |  |
| Childhood (0-12 years) | **0.23 (0.12-0.46), *P<*.001** | **0.13 (0.06-0.26), *P<*.001** | **0.27 (0.12-0.59), *P=*.001** | **0.25 (0.10-0.62), *P=*.003** |
| Adolescence (13-17 years) | **0.28 (0.16-0.51), *P<*.001** | **0.24 (0.13-0.43), *P<*.001** | **0.29 (0.15-0.56), *P<*.001** | **0.29 (0.14-0.61), *P=*.001** |
| Adulthood (18+ years) | 1.00 (Reference) | 1.00 (Reference) | 1.00 (Reference) | 1.00 (Reference) |
| **Gender identity** |  |  |  |  |
| Cisgender man | 0.92 (0.59-1.43), *P=*.708 | 1.08 (0.67-1.74), *P=*.750 | 0.95 (0.57-1.58), *P=*.830 | 0.96 (0.53-1.75), *P=*.904 |
| Transfeminine | 1.83 (0.55-6.16), *P=*.326 | 1.63 (0.44-6.07), *P=*.466 | 2.25 (0.55-9.21), *P=*.261 | 2.15 (0.42-11.07), *P=*.360 |
| Transmasculine | 0.96 (0.31-2.97), *P=*.950 | 0.56 (0.13-2.36), *P=*.426 | 0.61 (0.16-2.34), *P=*.468 | 0.32 (0.05-1.85), *P=*.202 |
| Nonbinary | 1.00 (0.50-2.00), *P=*.990 | **0.30 (0.10-0.94), *P=*.038** | 1.22 (0.52-2.85), *P=*.644 | 0.46 (0.12-1.83), *P=*.273 |
| Cisgender woman | 1.00 (Reference) | 1.00 (Reference) | 1.00 (Reference) | 1.00 (Reference) |
| **Racial identity** |  |  |  |  |
| Black/African American | **2.05 (1.12-3.76), *P=*.020** | **2.36 (1.26-4.44), *P=*.008** | 1.78 (0.89-3.56), *P=*.104 | 1.82 (0.83-3.98), *P=*.135 |
| Latine/Hispanic | 0.52 (0.23-1.19), *P=*.121 | 0.39 (0.13-1.11), *P=*.079 | 0.60 (0.23-1.55), *P=*.290 | 0.53 (0.15-1.87), *P=*.322 |
| Multiracial | 0.96 (0.43-2.12), *P=*.914 | 0.75 (0.28-1.97), *P=*.559 | 1.10 (0.44-2.75), *P=*.831 | 1.49 (0.46-4.90), *P=*.508 |
| Another racial identity | 0.71 (0.29-1.78), *P=*.467 | 0.54 (0.18-1.63), *P=*0.273 | 0.73 (0.26-2.08), *P=*0.560 | 0.51 (0.13-2.03), *P=*0.342 |
| White | 1.00 (Reference) | 1.00 (Reference) | 1.00 (Reference) | 1.00 (Reference) |
| **Sexual orientation other than heterosexual** |  |  |  |  |
| Yes | 0.85 (0.57-1.25), *P=*.406 | **0.46 (0.29-0.72), *P<*.001** | 0.76 (0.47-1.24), *P=*.279 | **0.50 (0.28-0.91), *P=*.023** |
| No | 1.00 (Reference) | 1.00 (Reference) | 1.00 (Reference) | 1.00 (Reference) |
| **Attained bachelor’s degree or higher** |  |  |  |  |
| Yes | **2.19 (1.47-3.28), *P<*.001** | **4.01 (2.49-6.48), *P<*.001** | 1.02 (0.62-1.69), *P=*.930 | 1.42 (0.76-2.65), *P=*.275 |
| No | 1.00 (Reference) | 1.00 (Reference) | 1.00 (Reference) | 1.00 (Reference) |
| **Currently unemployed** |  |  |  |  |
| Yes | **0.37 (0.20-0.69), *P=*.001** | **0.28 (0.13-0.62), *P=*.001** | 0.54 (0.26-1.09), *P=*.085 | 0.81 (0.30-2.21), *P=*.683 |
| No | 1.00 (Reference) | 1.00 (Reference) | 1.00 (Reference) | 1.00 (Reference) |
| **Self-reported community type** |  |  |  |  |
| Rural | **0.37 (0.19-0.70), *P=*.003** | **0.36 (0.17-0.74), *P=*.005** | 0.48 (0.22-1.04), *P=*.062 | 0.79 (0.31-2.02), *P=*.625 |
| Suburban | 0.81 (0.52-1.24), *P=*.331 | **0.55 (0.34-0.88), *P=*.014** | 0.92 (0.56-1.54), *P=*.762 | 0.70 (0.38-1.27), *P=*.242 |
| Urban | 1.00 (Reference) | 1.00 (Reference) | 1.00 (Reference) | 1.00 (Reference) |
| **U.S. Census region** |  |  |  |  |
| Midwest | 0.85 (0.47-1.55), *P=*.601 | 1.31 (0.70-2.48), *P=*.400 | 1.02 (0.51-2.02), *P=*.959 | 1.95 (0.88-4.34), *P=*.102 |
| Northeast | 0.96 (0.56-1.62), *P=*.866 | 1.10 (0.61-1.98), *P=*.751 | 1.14 (0.61-2.15), *P=*.679 | 1.18 (0.56-2.50), *P=*.665 |
| West | 1.29 (0.76-2.18), *P=*.343 | 1.22 (0.67-2.22), *P=*.518 | 1.47 (0.79-2.76), *P=*.226 | 1.23 (0.58-2.60), *P=*.584 |
| South | 1.00 (Reference) | 1.00 (Reference) | 1.00 (Reference) | 1.00 (Reference) |
| **TREATMENT HISTORY** |  |  |  |  |
| **Time from first symptoms to first receiving treatment** |  |  |  |  |
| 5+ years | 0.85 (0.51-1.41), *P=*.525 | 0.63 (0.36-1.12), *P=*.113 | 1.19 (0.66-2.14), *P=*.554 | 1.10 (0.55-2.22), *P=*.785 |
| 1-4 years | 0.70 (0.43-1.12), *P=*.136 | **0.42 (0.25-0.72), *P=*.001** | 0.80 (0.46-1.39), *P=*.433 | 0.57 (0.30-1.11), *P=*.098 |
| Less than one year | 1.00 (Reference) | 1.00 (Reference) | 1.00 (Reference) | 1.00 (Reference) |
| **Provider type for any received treatment** |  |  |  |  |
| **Medical practitioner (e.g., primary care physician)** |  |  |  |  |
| Yes | 0.93 (0.62-1.40), *P=*.742 | 0.66 (0.42-1.06), *P=*.086 | 0.91 (0.53-1.58), *P=*.739 | 0.68 (0.35-1.33), *P=*.259 |
| No | 1.00 (Reference) | 1.00 (Reference) | 1.00 (Reference) | 1.00 (Reference) |
| **Psychiatrist** |  |  |  |  |
| Yes | 1.20 (0.80-1.80), *P=*.378 | 0.83 (0.53-1.31), *P=*.430 | 1.31 (0.70-2.45), *P=*.405 | 1.20 (0.56-2.54), *P=*.638 |
| No | 1.00 (Reference) | 1.00 (Reference) | 1.00 (Reference) | 1.00 (Reference) |
| **Psychologist** |  |  |  |  |
| Yes | 1.07 (0.68-1.68), *P=*.769 | 1.36 (0.83-2.24), *P=*.224 | 0.91 (0.52-1.59), *P=*.743 | 1.45 (0.73-2.88), *P=*.287 |
| No | 1.00 (Reference) | 1.00 (Reference) | 1.00 (Reference) | 1.00 (Reference) |
| **Therapist** |  |  |  |  |
| Yes | 1.07 (0.72-1.60), *P=*.723 | 1.16 (0.74-1.82), *P=*.510 | 1.04 (0.57-1.90), *P=*.895 | **2.11 (1.00-4.41), *P=*.049** |
| No | 1.00 (Reference) | 1.00 (Reference) | 1.00 (Reference) | 1.00 (Reference) |
| **Types of treatment received** |  |  |  |  |
| **Medication** |  |  |  |  |
| Yes | 1.12 (0.73-1.73), *P=*.608 | 0.89 (0.56-1.43), *P=*.643 | 1.71 (0.78-3.76), *P=*.180 | 1.57 (0.64-3.84), *P=*.326 |
| No | 1.00 (Reference) | 1.00 (Reference) | 1.00 (Reference) | 1.00 (Reference) |
| **Individual therapy** |  |  |  |  |
| Yes | 1.31 (0.87-1.99), *P=*.198 | 1.15 (0.73-1.82), *P=*.542 | 0.98 (0.46-2.10), *P=*.967 | 0.84 (0.34-2.04), *P=*.692 |
| No | 1.00 (Reference) | 1.00 (Reference) | 1.00 (Reference) | 1.00 (Reference) |
| **Family/group therapy** |  |  |  |  |
| Yes | 0.92 (0.55-1.53), *P=*.749 | 1.16 (0.68-2.00), *P=*.585 | 0.62 (0.31-1.21), *P=*.160 | 0.61 (0.28-1.34), *P=*.219 |
| No | 1.00 (Reference) | 1.00 (Reference) | 1.00 (Reference) | 1.00 (Reference) |
| **Intensive outpatient program or partial hospitalization** |  |  |  |  |
| Yes | 0.68 (0.37-1.25), *P=*.211 | 0.63 (0.30-1.30), *P=*.211 | 0.52 (0.22-1.21), *P=*.131 | 0.52 (0.18-1.53), *P=*.236 |
| No | 1.00 (Reference) | 1.00 (Reference) | 1.00 (Reference) | 1.00 (Reference) |
| **Hospitalization** |  |  |  |  |
| Yes | 1.09 (0.65-1.84), *P=*.742 | 0.77 (0.40-1.47), *P=*.424 | 1.69 (0.77-3.73), *P=*.190 | 1.34 (0.49-3.66), *P=*.570 |
| No | 1.00 (Reference) | 1.00 (Reference) | 1.00 (Reference) | 1.00 (Reference) |
| **PHYSICAL AND PSYCHIATRIC COMORBIDITIES** |  |  |  |  |
| **Self-reported general health (item from SF-12)** |  |  |  |  |
| Poor/fair | 0.66 (0.43-1.00), *P=*.050 | **0.30 (0.17-0.52), *P<*.001** | 0.75 (0.46-1.24), *P=*.264 | **0.38 (0.19-0.74), *P=*.005** |
| Good/very good/excellent | 1.00 (Reference) | 1.00 (Reference) | 1.00 (Reference) | 1.00 (Reference) |
| **Number of psychiatric comorbidities** |  |  |  |  |
| 2+ | 1.01 (0.63-1.60), *P=*.982 | **0.42 (0.25-0.71), *P=*.001** | 1.14 (0.65-2.01), *P=*.643 | 0.67 (0.33-1.35), *P=*.262 |
| 1 | 1.07 (0.62-1.84), *P=*.798 | 0.65 (0.36-1.16), *P=*.143 | 1.44 (0.76-2.70), *P=*.261 | 0.90 (0.43-1.87), *P=*.770 |
| 0 | 1.00 (Reference) | 1.00 (Reference) | 1.00 (Reference) | 1.00 (Reference) |
| **PSYCHOSOCIAL FACTORS** |  |  |  |  |
| **Number of adverse childhood experiences (ACEs)** |  |  |  |  |
| 2+ | 0.74 (0.45-1.22), *P=*.236 | **0.38 (0.23-0.65), *P<*.001** | 0.97 (0.53-1.77), *P=*.923 | 0.73 (0.37-1.45), *P=*.375 |
| 1 | 1.18 (0.67-2.08), *P=*.557 | **0.54 (0.30-1.00), *P=*.050** | 1.48 (0.77-2.87), *P=*.240 | 1.01 (0.47-2.15), *P=*.978 |
| 0 | 1.00 (Reference) | 1.00 (Reference) | 1.00 (Reference) | 1.00 (Reference) |
| **Frequent engagement with an in-person or online social group** |  |  |  |  |
| Yes | **3.22 (2.05-5.08), *P<*.001** | **6.14 (3.73-10.10), *P<*.001** | **2.33 (1.37-3.97), *P=*.002** | **3.80 (2.05-7.03), *P<*.001** |
| No | 1.00 (Reference) | 1.00 (Reference) | 1.00 (Reference) | 1.00 (Reference) |
| **Perceived social support score, z-scored (items from MSPSS)** | **1.72 (1.39-2.13), *P<*.001** | **3.48 (2.56-4.74), *P<*.001** | **1.55 (1.20-1.99), *P<*.001** | **2.56 (1.80-3.64), *P<*.001** |

*Note*. All models incorporate a log-transformed offset term for a participant's number of years at risk for the outcome. Multivariable results are from a complete-case analysis. Within the analytic dataset (N=576), variable missingness ranged from 0 to 1.9% (MSPSS score). Bolding indicates statistical significance at $\alpha$ = .05. SF-12 = 12-Item Short Form Health Survey. MSPSS = Multidimensional Scale of Perceived Social Support.
