# Supplementary material for: Delays in diagnosis and treatment of depressive disorder among young adults: A national online survey-based cross-sectional study
Source: PLoS One. 2026 Jun 12;21(6):e0351402. doi: 10.1371/journal.pone.0351402 (PMC13262879; doi:10.1371/journal.pone.0351402)
Supplement: S3 Appendix — (DOCX) [file pone.0351402.s003.docx]

**S3 Appendix**. Perceived control of current depressive symptoms (N=237)

|  | **Bivariate models** | | **Multivariable model (N=232)** | |
| --- | --- | --- | --- | --- |
|  | **Moderately vs Slightly/not at all** | **Completely/well vs Slightly/not at all** | **Moderately vs Slightly/not at all** | **Completely/well vs Slightly/not at all** |
| **SOCIODEMOGRAPHICS** | **RRR (95% CI),**  **p-value** | **RRR (95% CI),**  **p-value** | **aRRR (95% CI),**  **p-value** | **aRRR (95% CI),**  **p-value** |
| **Age at first depression symptom onset** |  |  |  |  |
| Childhood (0-12 years) | 0.52 (0.19-1.40), *P=*.194 | **0.31 (0.11-0.86), *P=*.024** | 0.47 (0.14-1.58), *P=*.223 | 0.39 (0.11-1.45), *P=*.161 |
| Adolescence (13-17 years) | 0.71 (0.28-1.82), *P=*.479 | 0.59 (0.23-1.50), *P=*.269 | 0.98 (0.32-3.03), *P=*.971 | 0.73 (0.22-2.49), *P=*.620 |
| Adulthood (18+ years) | 1.00 (Reference) | 1.00 (Reference) | 1.00 (Reference) | 1.00 (Reference) |
| **Gender identity** |  |  |  |  |
| Cisgender man | 0.65 (0.31-1.37), *P=*.255 | 0.74 (0.35-1.57), *P=*.431 | 0.77 (0.30-1.96), *P=*.585 | 0.90 (0.32-2.52), *P=*.844 |
| Transfeminine | 1.40 (0.27-7.18), *P=*.685 | 0.63 (0.10-4.17), *P=*.635 | 1.28 (0.19-8.84), *P=*.801 | 0.89 (0.10-7.99), *P=*.918 |
| Transmasculine | 0.52 (0.10-2.77), *P=*.444 | 0.63 (0.12-3.38), *P=*.592 | 0.80 (0.10-6.20), *P=*.835 | 2.05 (0.23-18.08), *P=*.517 |
| Nonbinary | 0.93 (0.31-2.80), *P=*.890 | 0.43 (0.11-1.64), *P=*.216 | 0.78 (0.21-2.97), *P=*.720 | 0.68 (0.13-3.47), *P=*.647 |
| Cisgender woman | 1.00 (Reference) | 1.00 (Reference) | 1.00 (Reference) | 1.00 (Reference) |
| **Racial identity** |  |  |  |  |
| Black/African American | 1.12 (0.45-2.76), *P=*.805 | 1.06 (0.42-2.69), *P=*.906 | 1.18 (0.40-3.49), *P=*.765 | 0.90 (0.27-3.04), *P=*.872 |
| Latine/Hispanic | 1.10 (0.26-4.60), *P=*.900 | 0.93 (0.20-4.35), *P=*.932 | 2.39 (0.38-15.04), *P=*.355 | 0.99 (0.11-8.63), *P=*.994 |
| Multiracial | 0.71 (0.20-2.48), *P=*.588 | 0.71 (0.19-2.63), *P=*.612 | 0.60 (0.14-2.54), *P=*.488 | 0.58 (0.12-2.73), *P=*.491 |
| Another racial identity | 0.59 (0.14-2.56), *P=*.483 | 0.40 (0.07-2.23), *P=*.298 | 0.68 (0.11-4.04), *P=*.669 | 0.31 (0.04-2.53), *P=*.273 |
| White | 1.00 (Reference) | 1.00 (Reference) | 1.00 (Reference) | 1.00 (Reference) |
| **Sexual orientation other than heterosexual** |  |  |  |  |
| Yes | 1.33 (0.69-2.56), *P=*.395 | 0.52 (0.26-1.04), *P=*.065 | 1.38 (0.62-3.09), *P=*.428 | 0.77 (0.32-1.88), *P=*.570 |
| No | 1.00 (Reference) | 1.00 (Reference) | 1.00 (Reference) | 1.00 (Reference) |
| **Attained bachelor’s degree or higher** |  |  |  |  |
| Yes | **2.41 (1.23-4.73), *P=*.010** | **2.93 (1.44-5.96), *P=*.003** | 2.37 (0.94-5.98), *P=*.068 | 1.25 (0.45-3.47), *P=*.665 |
| No | 1.00 (Reference) | 1.00 (Reference) | 1.00 (Reference) | 1.00 (Reference) |
| **Currently unemployed** |  |  |  |  |
| Yes | 0.51 (0.20-1.29), *P=*.154 | **0.31 (0.10-0.95), *P=*.040** | 0.54 (0.16-1.90), *P=*.340 | 0.40 (0.09-1.86), *P=*.244 |
| No | 1.00 (Reference) | 1.00 (Reference) | 1.00 (Reference) | 1.00 (Reference) |
| **Self-reported community type** |  |  |  |  |
| Rural | 1.51 (0.57-4.01), *P=*.408 | 0.59 (0.20-1.80), *P=*.357 | 1.93 (0.57-6.49), *P=*.289 | 0.74 (0.18-3.08), *P=*.679 |
| Suburban | **2.19 (1.06-4.52), *P=*.035** | 1.32 (0.63-2.76), *P=*.456 | **2.64 (1.10-6.36), *P=*.030** | 1.17 (0.46-3.02), *P=*.739 |
| Urban | 1.00 (Reference) | 1.00 (Reference) | 1.00 (Reference) | 1.00 (Reference) |
| **U.S. Census region** |  |  |  |  |
| Midwest | 0.59 (0.25-1.42), *P=*.242 | **0.20 (0.06-0.67), *P=*.009** | 0.53 (0.17-1.61), *P=*.261 | **0.19 (0.04-0.79), *P=*.023** |
| Northeast | 0.92 (0.38-2.20), *P=*.843 | 1.19 (0.49-2.88), *P=*.698 | 1.31 (0.44-3.90), *P=*.624 | 1.41 (0.44-4.54), *P=*.565 |
| West | 1.21 (0.46-3.18), *P=*.696 | 1.53 (0.58-4.04), *P=*.390 | 1.75 (0.51-6.00), *P=*.373 | 2.09 (0.56-7.76), *P=*.272 |
| South | 1.00 (Reference) | 1.00 (Reference) | 1.00 (Reference) | 1.00 (Reference) |
| **TREATMENT HISTORY** |  |  |  |  |
| **Time from first symptoms to first receiving treatment** |  |  |  |  |
| 5+ years | 1.18 (0.52-2.67), *P=*.687 | 0.69 (0.30-1.60), *P=*0.385 | 1.80 (0.63-5.19), *P=*0.274 | 1.40 (0.45-4.39), *P=*0.563 |
| 1-4 years | 1.01 (0.45-2.27), *P=*.972 | 0.68 (0.30-1.54), *P=*0.351 | 1.14 (0.42-3.07), *P=*0.793 | 0.95 (0.31-2.88), *P=*0.929 |
| Less than one year | 1.00 (Reference) | 1.00 (Reference) | 1.00 (Reference) | 1.00 (Reference) |
| **Provider type for any received treatment** |  |  |  |  |
| **Medical practitioner (e.g., primary care physician)** |  |  |  |  |
| Yes | 1.16 (0.58-2.31), *P=*.667 | 0.52 (0.26-1.04), *P=*.065 | 1.05 (0.44-2.46), *P=*.919 | 0.74 (0.29-1.85), *P=*.517 |
| No | 1.00 (Reference) | 1.00 (Reference) | 1.00 (Reference) | 1.00 (Reference) |
| **Psychiatrist** |  |  |  |  |
| Yes | 0.59 (0.28-1.26), *P=*.176 | 0.60 (0.28-1.31), *P=*.201 | 0.44 (0.15-1.29), *P=*.136 | 0.96 (0.29-3.14), *P=*.942 |
| No | 1.00 (Reference) | 1.00 (Reference) | 1.00 (Reference) | 1.00 (Reference) |
| **Psychologist** |  |  |  |  |
| Yes | 0.95 (0.48-1.89), *P=*.881 | 1.18 (0.58-2.39), *P=*.653 | 0.75 (0.32-1.74), *P=*.500 | 1.26 (0.49-3.22), *P=*.629 |
| No | 1.00 (Reference) | 1.00 (Reference) | 1.00 (Reference) | 1.00 (Reference) |
| **Therapist** |  |  |  |  |
| Yes | 1.14 (0.58-2.25), *P=*.704 | 0.83 (0.42-1.67), *P=*.611 | 1.10 (0.45-2.70), *P=*.840 | 1.11 (0.41-2.99), *P=*.840 |
| No | 1.00 (Reference) | 1.00 (Reference) | 1.00 (Reference) | 1.00 (Reference) |
| **Types of treatment received** |  |  |  |  |
| **Medication** |  |  |  |  |
| Yes | 0.93 (0.26-3.37), *P=*.911 | 0.71 (0.20-2.51), *P=*.600 | 1.34 (0.23-7.82), *P=*.743 | 0.91 (0.14-6.15), *P=*.926 |
| No | 1.00 (Reference) | 1.00 (Reference) | 1.00 (Reference) | 1.00 (Reference) |
| **Individual therapy** |  |  |  |  |
| Yes | 0.96 (0.36-2.58), *P=*.936 | 0.60 (0.23-1.57), *P=*.298 | 0.87 (0.21-3.62), *P=*.849 | 0.26 (0.06-1.20), *P=*.084 |
| No | 1.00 (Reference) | 1.00 (Reference) | 1.00 (Reference) | 1.00 (Reference) |
| **Family/group therapy** |  |  |  |  |
| Yes | 0.55 (0.26-1.16), *P=*.117 | 0.72 (0.34-1.52), *P=*.386 | 0.52 (0.19-1.43), *P=*.203 | 0.36 (0.12-1.09), *P=*.072 |
| No | 1.00 (Reference) | 1.00 (Reference) | 1.00 (Reference) | 1.00 (Reference) |
| **Intensive outpatient program or partial hospitalization** |  |  |  |  |
| Yes | 0.54 (0.24-1.24), *P=*.145 | 0.45 (0.18-1.12), *P=*.086 | 0.39 (0.12-1.32), *P=*.129 | 0.50 (0.13-1.96), *P=*.321 |
| No | 1.00 (Reference) | 1.00 (Reference) | 1.00 (Reference) | 1.00 (Reference) |
| **Hospitalization** |  |  |  |  |
| Yes | 1.06 (0.50-2.22), *P=*.881 | 0.73 (0.32-1.63), *P=*.436 | **3.82 (1.15-12.69), *P=*.029** | 3.11 (0.81-11.91), *P=*.098 |
| No | 1.00 (Reference) | 1.00 (Reference) | 1.00 (Reference) | 1.00 (Reference) |
| **PHYSICAL AND PSYCHIATRIC COMORBIDITIES** |  |  |  |  |
| **Self-reported general health (item from SF-12)** |  |  |  |  |
| Poor/fair | **0.43 (0.22-0.84), *P=*.014** | **0.19 (0.09-0.41), *P<*.001** | 0.46 (0.21-1.02), *P=*.056 | **0.19 (0.08-0.49), *P<*.001** |
| Good/very good/excellent | 1.00 (Reference) | 1.00 (Reference) | 1.00 (Reference) | 1.00 (Reference) |
| **Number of psychiatric comorbidities** |  |  |  |  |
| 2+ | 1.00 (0.47-2.14), *P=*.997 | 0.64 (0.30-1.38), *P=*.259 | 0.94 (0.36-2.43), *P=*.890 | 0.73 (0.25-2.13), *P=*.561 |
| 1 | 0.93 (0.35-2.45), *P=*.886 | 0.56 (0.20-1.53), *P=*.258 | 0.83 (0.25-2.72), *P=*.762 | 0.55 (0.15-2.06), *P=*.375 |
| 0 | 1.00 (Reference) | 1.00 (Reference) | 1.00 (Reference) | 1.00 (Reference) |
| **PSYCHOSOCIAL FACTORS** |  |  |  |  |
| **Number of adverse childhood experiences (ACEs)** |  |  |  |  |
| 2+ | 0.91 (0.36-2.30), *P=*.848 | **0.27 (0.11-0.65), *P=*.004** | 1.27 (0.40-3.99), *P=*.682 | 0.34 (0.10-1.11), *P=*.075 |
| 1 | 1.15 (0.41-3.26), *P=*.791 | 0.41 (0.15-1.13), *P=*.085 | 1.93 (0.55-6.78), *P=*.306 | 0.67 (0.18-2.43), *P=*.539 |
| 0 | 1.00 (Reference) | 1.00 (Reference) | 1.00 (Reference) | 1.00 (Reference) |
| **Frequent engagement with an in-person or online social group** |  |  |  |  |
| Yes | 1.42 (0.72-2.82), *P=*.309 | **2.18 (1.07-4.43), *P=*.031** | 1.44 (0.61-3.42), *P=*.410 | 1.45 (0.53-3.92), *P=*.466 |
| No | 1.00 (Reference) | 1.00 (Reference) | 1.00 (Reference) | 1.00 (Reference) |
| **Perceived social support score, z-scored (items from MSPSS)** | **1.55 (1.12-2.14), *P=*.008** | **2.74 (1.81-4.14), *P<*.001** | 1.17 (0.78-1.77), *P=*.451 | **2.36 (1.39-3.98), *P=*.001** |

*Note*. All models incorporate a log-transformed offset term for a participant's number of years at risk for the outcome. Multivariable results are from a complete-case analysis. Within the analytic dataset (N=237), variable missingness ranged from 0 to 1.3% (MSPSS score). Bolding indicates statistical significance at $\alpha$ = .05. SF-12 = 12-Item Short Form Health Survey. MSPSS = Multidimensional Scale of Perceived Social Support.
