# Supplementary material for: Delays in diagnosis and treatment of depressive disorder among young adults: A national online survey-based cross-sectional study
Source: PLoS One. 2026 Jun 12;21(6):e0351402. doi: 10.1371/journal.pone.0351402 (PMC13262879; doi:10.1371/journal.pone.0351402)
Supplement: S4 Appendix — (DOCX) [file pone.0351402.s004.docx]

**S4 Appendix. Sensitivity analysis excluding participants with bipolar disorder diagnosis**





Dashed vertical line = null (1.0); aPR = adjusted prevalence ratio (modified Poisson with robust SEs); aRR = adjusted rate ratio (negative binomial); aRRR = adjusted relative risk ratio (multinomial logistic). Multinomial outcome contrasts appear in brackets at the start of each. An asterisk (*) flags a covariate that was significant in the primary model but lost significance after excluding bipolar participants.
